# Cigarette side-stream smoke lung and bladder carcinogenesis: inducing mutagenic acrolein-DNA adducts, inhibiting DNA repair and enhancing anchorage-independent-growth cell transformation

**DOI:** 10.18632/oncotarget.5429

**Published:** 2015-09-28

**Authors:** Hyun-Wook Lee, Hsiang-Tsui Wang, Mao-wen Weng, Chiu Chin, William Huang, Herbert Lepor, Xue-Ru Wu, William N. Rom, Lung-Chi Chen, Moon-shong Tang

**Affiliations:** ^1^ Department of Environmental Medicine, New York University School of Medicine, New York, NY, USA; ^2^ Department of Urology, New York University School of Medicine, New York, NY, USA; ^3^ Department of Medicine, New York University School of Medicine, New York, NY, USA; ^4^ Department of Pathology, New York University School of Medicine, New York, NY, USA

**Keywords:** second-hand and side-stream smoke, acrolein and BPDE, DNA damage and repair, lung and bladder cancer, anchorage independent growth

## Abstract

Second-hand smoke (SHS) is associated with 20–30% of cigarette-smoke related diseases, including cancer. Majority of SHS (>80%) originates from side-stream smoke (SSS). Compared to mainstream smoke, SSS contains more tumorigenic polycyclic aromatic hydrocarbons and acrolein (Acr). We assessed SSS-induced benzo(a)pyrene diol epoxide (BPDE)- and cyclic propano-deoxyguanosine (PdG) adducts in bronchoalveolar lavage (BAL), lung, heart, liver, and bladder-mucosa from mice exposed to SSS for 16 weeks. In SSS exposed mice, Acr-dG adducts were the major type of PdG adducts formed in BAL (*p* < 0.001), lung (*p* < 0.05), and bladder mucosa (*p* < 0.001), with no significant accumulation of Acr-dG adducts in heart or liver. SSS exposure did not enhance BPDE-DNA adduct formation in any of these tissues. SSS exposure reduced nucleotide excision repair (*p* < 0.01) and base excision repair (*p* < 0.001) in lung tissue. The levels of DNA repair proteins, XPC and hOGG1, in lung tissues of exposed mice were significantly (*p* < 0.001 and *p* < 0.05) lower than the levels in lung tissues of control mice. We found that Acr can transform human bronchial epithelial and urothelial cells *in vitro*. We propose that induction of mutagenic Acr-DNA adducts, inhibition of DNA repair, and induction of cell transformation are three mechanisms by which SHS induces lung and bladder cancers.

## INTRODUCTION

Cigarette smoke (CS) is the major cause of human cancer, with more than 5 million CS-related cancer deaths worldwide annually [1]. Numerous studies have unambiguously established the relationship between mainstream smoke (MSS) and human cancer [2–7]. Many epidemiology studies suggest that second hand smoke (SHS) is responsible for 20–30% of CS related diseases, including cancer [7–12]. These findings underscore the importance of establishing CS-free environments in order to reduce human cancer.

More than 80% of SHS originates from side stream smoke (SSS) [13]. Many studies have found that on a per total particulate matter basis, SSS is two- to five-fold more toxic and mutagenic than mainstream smoke (MSS) [13–15]. The underlying mechanisms of what causes SSS to be more toxic and mutagenic than MSS are unclear. Compared with MSS, SSS contains more polycyclic aromatic hydrocarbons (PAHs) and aldehydes, such acrolein (Acr), formaldehyde, and acetaldehyde [13, 15]. Metabolically activated PAHs can induce mutagenic DNA damage [16, 17]. Acr can both induce mutagenic DNA adducts and modify repair proteins, causing DNA repair dysfunction and degradation [18–21]. Acr also enhances the susceptibility of cells to mutagenesis [18, 20, 21]. These findings raise the possibility that induction of DNA damage by PAHs and Acr and inhibition of DNA repair by Acr are two mechanisms by which SSS causes toxic, mutagenic, and perhaps tumorigenic effects. We tested this possibility by determining the levels of two major classes of DNA adduct induced by PAHs and aldehydes: benzo(a)pyrene diol epoxide (BPDE)-DNA adducts and aldehyde-induced cyclic propano-deoxyguanosine (PdG) adducts in bronchoalveolar lavage (BAL), lung, heart, liver, and bladder-mucosa tissues from mice exposed to SSS for 8 and 16 weeks. We also determined the effect of SSS on nucleotide excision repair (NER) and base excision repair (BER) in lung tissues, as well as the effect of Acr on tumorigenic cell transformation activity in human bronchial epithelial and urothelial cells. We found that SSS induced mutagenic Acr-DNA adducts and inhibited DNA repair. Acr also induced cell transformation *in vitro*. We propose that these are the three mechanisms by which SHS causes lung and bladder cancers.

## RESULTS

### Exposure of mice to SSS

SSS was generated from a smoke machine by burning one cigarette at a time at a burning rate of one cigarette per 12 min. The 500 μg/m^3^ of smoke particulate matters generated in the SSS is roughly equivalent to the smoke concentration in a household of a habitual heavy smoker [22]. A total of 39 male mice (9–10 mice/group) was either exposed to SSS or filtered air (FA) 6 h/day and 5 days/week for 8 and 16 weeks. At the end of exposure mice were sacrificed, the bronchoalveolar lavage (BAL) was collected, and lung, heart, liver, and bladder-mucosa tissues were harvested and snap frozen at −80°C. For DNA adduct analysis, genomic DNAs were prepared from the BAL, lung, heart, liver, and mucosa of bladder tissues [20]. For the DNA repair assay, cell lysates were prepared from lung tissues of mice exposed to SSS or FA [18, 19, 23]. During the 8- and 16-week exposure period, all mice appeared to be healthy (no weight lost, fur normal, and no unusual behavior), and all the mice survived until after the end of experimental period.

### SSS induces Acr-DNA adducts in lung and bladder

**Figure 1:**
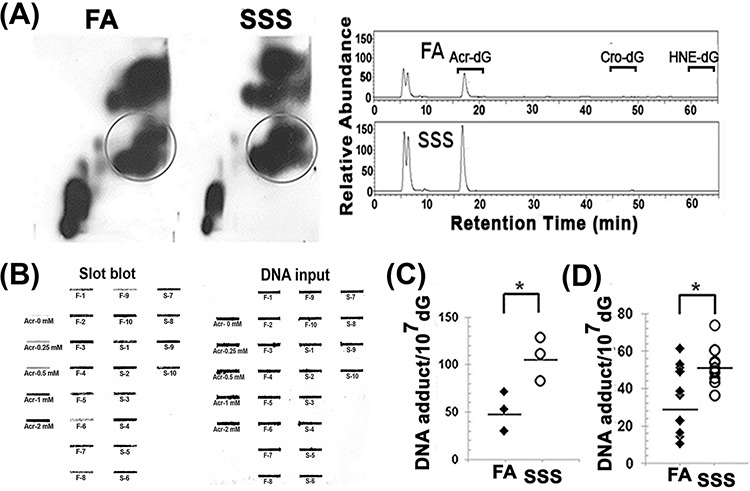
The acrolein-deoxyguanosine (Acr-dG) adduct is the major cyclic propano-DNA adduct formed in the lung tissues of mice exposed to side-stream smoke Genomic DNAs were isolated from the lung tissues of mice exposed to filtered air (FA) and side-stream smoke (SSS) for 16 weeks. Lung genomic DNA from three randomly chosen mice from the same treatment group were pooled together as one set (a total of three sets) for PdG adduct analysis by the ^32^P-post-labeling and 2D-TLC/HPLC method [20]. **A.** Left panels are typical 2D-TLC autoradiographs of FA and SSS samples. The spots with circles were eluted from the TLC plates and subjected to further analysis by HPLC. Right panels show typical HPLC elution profiles. The elution positions of the Acr-dG, HNE-dG, and Cro-dG adducts are indicated. PdG adducts formed in the lung genomic DNA were also analyzed by the immunochemical slot blot method, as described previously [18, 20]. **B.** A typical slot blot result. Left panel: fluorescent slot blot results. Right panel: input DNA stained by methylene blue. The genomic DNAs modified with different concentrations of Acr (Acr-0 mM to Acr-2 mM) were used as standards for quantitation. F-1 to F-10 represent genomic DNA isolated from lung tissues of mice exposed to filtered air for 16 weeks. S-1 to S-10 represent genomic DNA isolated from lung tissues of mice exposed to side-stream smoke for 16 weeks. The quantitative results of PdG formation in lung tissues are shown using both the 2D-TLC/HPLC method **C.** and the immunochemical method **D.** Bars represent the geometric median levels of PdG adducts. * represent *P* values of < 0.05.

CS is the major cause of human cancer, particularly lung and bladder cancer [24–28]. SSS is rich in Acr and PAHs, which can induce mutagenic DNA adducts [16–18, 20, 29]. Benzo(a)pyrene (BP) is one of the most mutagenic and tumorigenic PAHs found in CS [30–32]. BP induces mainly BPDE-DNA adducts in both cultured human cells and animal models [33–37]. The major DNA adducts induced by Acr are γ-OH-*1,N^2^*-propano-dG (Acr-dG) adducts, which have been found to be as mutagenic as BPDE-DNA adducts [21, 29, 38, 39]. The distribution of DNA adducts induced by these two compounds in the p53 gene of human bronchial epithelial cells are similar, if not identical, to the p53 mutational spectrum in human lung cancer [19, 40, 41]. These two compounds have been proposed to be important human lung cancer etiological agents in CS and cooking fumes [19, 21, 40, 42]. For these reasons we determined the levels of BPDE- and Acr-DNA adducts in the BAL, lung, bladder-mucosa, heart, and liver of mice exposed to SSS and FA. Acr-DNA adducts in the genomic DNA isolated from lung tissues were first determined by the well-established 2D-TLC and HPLC analysis method [18–21]; this method is able to determine the cyclic propano-dG induced by Acr, acetaldehyde (Acet), crotonaldehyde (Cro), and 4-hydroxy-2′-nonenal (HNE), which are abundant in CS [21, 25, 43–45]. The results in Fig. 1A show that the major cyclic propano-dG adducts (PdGs) formed in lung of mice exposed to SSS for 16 weeks co-chromatographed with Acr-dG adducts. DNA adducts formed in lung tissues of mice exposed to SSS also reacted with a monoclonal antibody against Acr-dG adducts (Fig. 1B). Therefore, we conclude that SSS exposure induces Acr-dG adducts. These results are consistent with the finding that Acr is a major aldehyde in SSS [13, 46]. Both the 2D-TLC/HPLC and the immunochemical method yielded similar, nearly identical, results (Fig. 1C and 1D).

**Figure 2:**
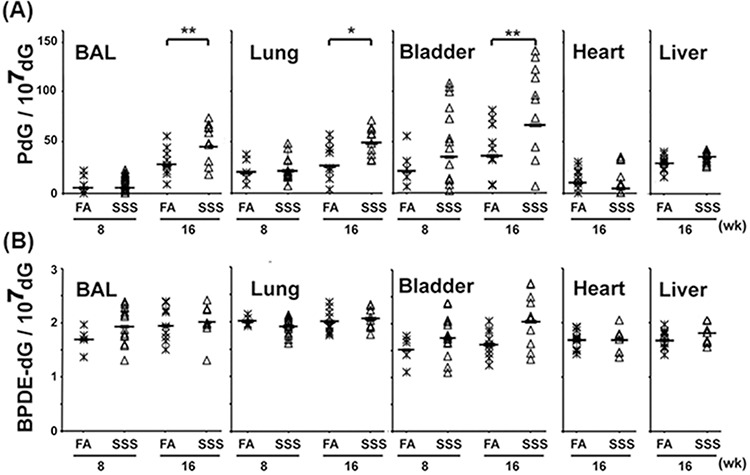
Acr-dG adducts are induced in the BAL, lung, and bladder-mucosa but not in the heart and liver of mice exposed to side-stream smoke (SSS). In contrast, SSS does not affect the formation of BPDE-dG adducts in any of these tissues Genomic DNAs isolated from BAL and the lung, heart, liver, and bladder mucosa from mice exposed to SSS [*n* = 10, except for BAL, lung, and bladder-mucosa from the 8-week exposure where *n* = 14] and filtered air (FA) [*n* = 10, except for BAL, lung, bladder mucosa from the 8-week exposure where *n* = 5] for 8 and 16 weeks were analyzed for the formation of cyclic PdG and BPDE-dG adducts by the immunochemical method described in Fig. 1 [18, 20]. Bars represent the geometric median levels of PdG **A.** and BPDE-dG **B.** adducts. * and ** represent *P* values of < 0.05 and < 0.01, respectively. Note: no statistical significance in PdG formation was found in 1) lung samples from mice with 8-week exposure of SSS and FA exposure (*p* = 0.9), 2) lung samples from mice exposed to FA for 8 weeks and 16 weeks (*p* = 0.26), and 3) bladder samples from mice exposed to FA for 8 weeks and 16 weeks (*p* = 0.12).

Owing to the very limited amount of genomic DNA (<1 μg) isolated from the BAL and bladder mucosa for each mouse, the Acr-dG adduct levels in these tissues were assessed only by the immunochemical method, using the monoclonal antibody against Acr-dG adducts [20, 29]. Fig. 2A shows that exposure to SSS affects the Acr-dG levels in the BAL, lung, and bladder mucosa, but not the liver or heart of mice. Although Acr-dG levels in the BAL, lung, and bladder mucosa in mice exposed to SSS or FA for 8 weeks were not significantly different (*p* = 0.49, *p* = 0.44 and *p* = 0.07 respectively), Acr-dG levels in the BAL, lung, and bladder mucosa in mice exposed to SSS for 16 weeks were twice those from mice exposed to FA. In contrast, Acr-dG levels in liver and heart tissues were the same for mice exposed to SSS and FA for 16 weeks.

### SSS does not induce BPDE-DNA adducts in lung, heart, liver, and bladder-mucosa

BPDE-DNA adducts levels in the genomic DNA isolated from the BAL, lung, liver, heart, and bladder-mucosa were analyzed by an immunochemical method, using a monoclonal antibody against BPDE-dG adducts [20]. The results in Fig. 2B show that: 1) the levels of BPDE-DNA adducts in the BAL, lung, liver, heart, and bladder-mucosa show no significant differences between mice exposed to SSS and to FA for 16 weeks (*p* = 0.32, *p* = 0.1, *p* = 0.23, *p* = 0.98 and *p* = 0.1 respectively); 2) the levels of BPDE-DNA adducts in the BAL, lung, bladder-mucosa, liver, and heart are 20–40 fold lower than Acr-dG adduct levels in mice exposed to SSS for 16 weeks; and 3) the extent of variations of BPDE-dG adducts among different mice with and without exposed to SSS is smaller than those of the Acr-dG levels.

### SSS exposure causes a reduction of NER and BER capacity

**Figure 3:**
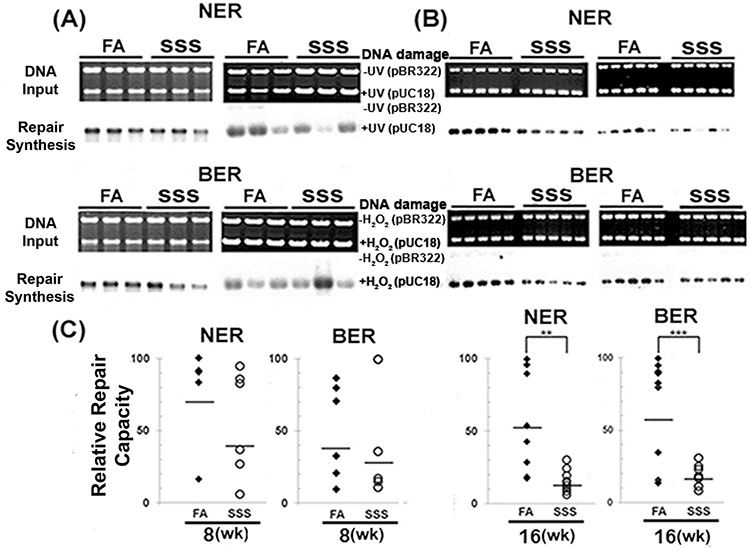
Side-stream smoke inhibits DNA repair Cell lysates were isolated from lung tissues of mice (*n* = 6 for 8 weeks, *n* = 10 for 16 weeks) exposed to side-stream smoke (SSS) or filtered air (FA) for 8 (A & C) or 16 (B & C) weeks. **A and B.** The NER and BER capacities in these lysates were determined using the DNA-damage-dependent repair synthesis method as previously described [19, 20]. UV-irradiated and H_2_O_2_-modified DNA substrates were used to measure NER and BER capacity. Upper panel: input DNA stained using ethidium bromide. Lower panel: autoradiographic bands. The band intensity represents the level of DNA repair synthesis. Relative NER and BER capacities shown in **C.** were calculated based on comparison of the intensity of each band to the band with the highest intensity (arbitrary assigned with *a* value of 100). ** and *** represent *P* values of < 0.01 and < 0.001, respectively. Note: The NER and BER activity in the lung tissues of mice exposed to SSS were lower than the activity in the lung tissues of mice exposed to FA.

The previous results indicate that Acr induces cyclic PdG DNA adducts in the BAL, lung, and bladder-mucosa of SSS-exposed mice. We have reported that Acr exposure inhibits DNA repair, including nucleotide excision repair (NER) and base excision repair (BER), in cultured human bronchial epithelial cells and urothelial cells, and consequently enhances the mutational susceptibility of the exposed cells [18–20]. These results raise the possibility that SSS can inhibit NER and BER in lung and bladder tissues and enhance the susceptibility of the exposed cells to mutagenesis. To test this possibility, we used an *in vitro* DNA-dependent repair synthesis method to determine the NER and BER activity in cell lysates isolated from lung tissues of mice exposed to SSS and FA. It was not possible to obtain adequate amounts of cell lysate from the bladder-mucosa to conduct this assay. Results in Fig. 3A–3C show that: 1) the activity of both NER and BER showed a wide range in mice exposed to FA; 2) the variation of NER and BER in 16-week-SSS-exposed mice were much smaller than those in FA exposed mice; and 3) most importantly, the NER and BER activity in lung tissues of mice exposed to SSS for 8 and 16 weeks were lower than for their counterparts of FA exposed mice, with the effect being more profound in mice exposed for 16 weeks (*p* < 0.01 and *p* < 0.001 for NER and BER) than in mice exposed for 8 weeks.

### SSS decreases levels of repair proteins

**Figure 4:**
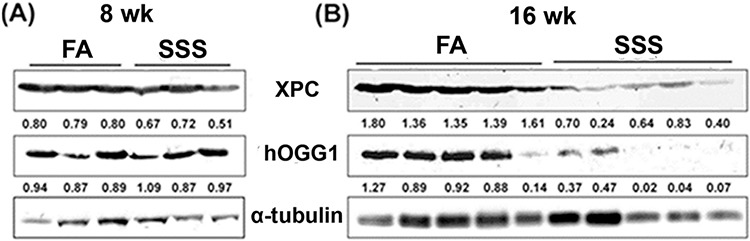
Side-stream smoke reduces the levels of XPC and hOGG1 repair proteins XPC and hOGG1 in cell lysates isolated from lung tissues of mice exposed to side-stream smoke (SSS; *n* = 3 for 8 weeks, *n* = 5 for 16 weeks) or filtered air (FA; *n* = 3 for 8 weeks, *n* = 5 for 16 weeks) for 8 or 16 weeks were detected by the method previously described [18, 20]. The relative levels of XPC and hOGG1 normalized to α-tubulin are indicated. Note: The levels of XPC (a NER protein) and hOGG1 (a BER protein) in the lung tissues of mice exposed to SSS for 16 weeks are lower than in the lung tissues of mice exposed to FA (*p* = 0.0002 and *p* = 0.02, for XPC and hOGG1, respectively).

Previously we have found that Acr can modify DNA repair proteins causing DNA repair dysfunction and autophagy-dependent DNA repair protein degradation in cultured human bronchial epithelial cells and urothelial cells [18, 20, 47]. Given both that Acr is a major component of SSS and our demonstration that BAL, bladder-mucosa and lung tissues of SSS-exposed mice have higher levels of Acr-dG adducts than those exposed to FA, these results raise the possibility that Acr in SSS can modify DNA repair proteins, thereby causing the degradation of these proteins. If this is the case, then we expect that the levels of some of the NER and BER proteins in lung tissues in SSS-exposed mice are lower than those in FA-exposed control mice. Results in Figs. 4A and 4B show that the levels of XPC and hOGG1 in lung tissues of mice exposed to SSS for 16 weeks are substantially lower (*p* = 0.0002 and *p* = 0.02, for XPC and hOGG1, respectively) than those of FA-exposed mice, although 8 weeks of SSS exposure does not cause this effect. XPC is a key NER component for genomic DNA overall [48] and hOGG1 is a BER enzyme, which has glycosylase and phosphodiesterase functions to repair 8-oxo-dG, a major product of oxidative damage to DNA [49]

### Acr induces tumorigenic cell transformation

**Figure 5:**
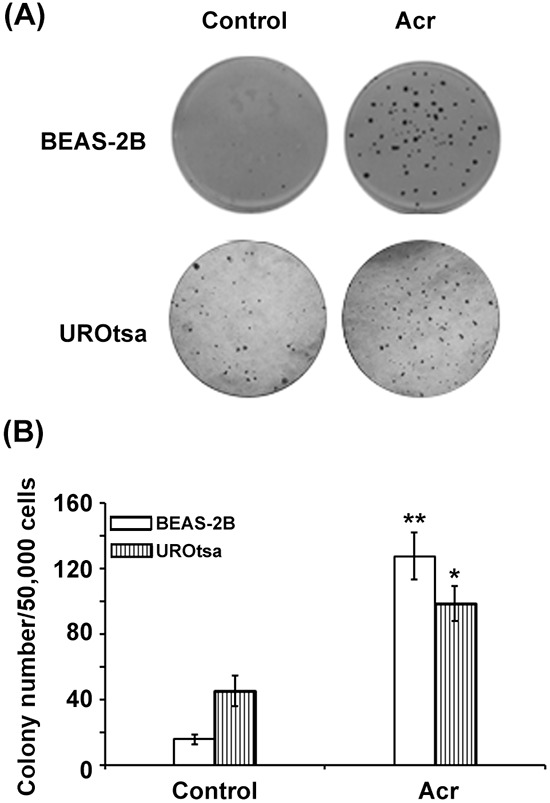
Acrolein induces tumorigenic transformation in human bronchial epithelial and urothelial cells Immortalized human bronchial epithelial cells BEAS-2B and human urothelial cells UROtsa were treated with Acr (2.5 μM) for 1 h, 50,000 of treated cells and untreated were seeded (duplicated) in a 6-cm dish with soft-agar growth medium as described [56]. The ability to grow in soft-agar was assessed by the formation of number of colonies with more than 50 cells/colony [56]. **A.** Typical results of soft agar growth of Acr-treated BEAS-2B and UROtsa cells. **B.** Number of colony formed in the soft-agar medium in BEAS-2B and UROtsa cells with and without Acr treatment. * and ** represent *P* values of < 0.05 and < 0.01, respectively.

The results presented above show that SSS induces Acr-dG adducts in BAL, lung tissues and bladder- mucosa, and inhibits NER and BER and reduces the levels of XPC and hOGG1 repair proteins in lung tissues. These results suggest that Acr is a major component in SSS responsible for inducing human lung and bladder cancer. Although the mutagenicity of Acr-dG has been established, the tumorigenicity of Acr has not been unambiguously demonstrated in animal models [21, 50, 51]. Although mice are extremely sensitive to Acr exposure following inhalation, the mechanisms that underlie the Acr lethality in mice remain unclear [52]. As a first step to establishing the tumorigenicity of Acr in lung and bladder, we determined the ability of Acr to induce anchorage-independent growth (a hallmark of neoplastic cell transformation) of immortalized human bronchial epithelial cells BEAS-2B and urothelial cells UROtsa [53–55]. Cells were treated with Acr (2.5 μM of Acr for 1 h at 37°C) and the effect of Acr on cell anchorage-independent growth was then assessed by growth in soft agar [53, 56]. The results in Fig. 5 show that Acr treatment induced significant anchorage-independent growth in both bronchial epithelial cells (*p* < 0.01) and urothelial cells (*p* < 0.05). These results indicate that Acr can cause tumorigenic transformation in both human bronchial epithelial and urothelial cells.

## DISCUSSION

Tobacco smoke is the major cause of human cancer; this conclusion is mainly derived from smokers have a higher cancer incidence than the general population, that main stream smoke contains a variety of human carcinogens, and that MSS causes cancer in animal models [26, 57–60]. In contrast, although it is generally believed that SHS is hazardous to human health, including the induction of cancer, direct biochemical evidence of SHS on carcinogenic mechanisms is lacking [61]. The Clean Air Act that banned smoking in public settings, is mainly based on epidemiological studies and the findings that tobacco carcinogens in children's urines of smokers’ households [62–64]. The current study presents evidence that SSS induces mutagenic Acr-dG adducts, inhibits DNA repair, and causes degradation of DNA repair proteins. We also show that Acr, the major aldehydes in SSS, can induce tumorigenic cell transformation.

Although SSS is thought to be more toxic and mutagenic than MSS, this conclusion is derived primarily from results obtained using smoke that was collected from SSS and stored [13, 26]. Given that chemical reactions may occur during SSS condensation and storage, those results might not reflect the effect of SSS immediately after it is generated [13, 26]. To avoid this potential complication, we used SSS generated in real-time to address its actual effects on DNA adduct formation and DNA repair capacity.

Both MSS and SSS contain many aldehydes, including formaldehyde, acetaldehyde, crotonaldehyde, and Acr [26]. CS contains higher levels of acetaldehyde and formaldehyde than Acr [26]; however, we found that SSS induces mainly Acr-dG adducts, rather than acetaldehyde- or crotonaldehyde-induced DNA adducts in BAL, lung, and bladder mucosa of SSS-exposed mice (Fig. 1). Acr differs from most aldehydes insofar as it can be readily absorbed by human cells [19–21, 65]. Given that the boiling temperature of acetaldehyde is much lower than that of most aldehydes at ambient temperature [66], the bioavailability of acetaldehyde is probably much lower than that of other aldehydes. These factors together may contribute to the fact that only Acr-induced DNA adducts are observed in the BAL, lung, and bladder mucosa of SSS-exposed mice.

It is intriguing that SSS inhalation causes an increase in Acr-dG adduct formation in BAL, lung, and bladder mucosa but not in heart and liver. Given that respiratory organs are the main paths for inhaled SSS, bronchial epithelial cells are exposed to more SSS than cells in other organs. Glutathione in lung cells can effectively conjugate with Acr and the conjugated Acr is then excreted [65]. Therefore, it is possible that once the level of inhaled Acr overwhelms the level of cellular glutathione, the unconjugated Acr may react with other cellular components, including DNA, to form adducts. This is probably the major reason that, when compared with mice exposed to FA, levels of Acr-dG adducts in BAL, lung, and bladder-tissues are significantly higher in mice exposed to SSS for 16 weeks but not for 8 weeks. We expect that once Acr enters into the bloodstream it reacts quickly with proteins, and the protein- and glutathione-conjugated Acr in the bloodstream are unable to enter other organs, such as liver and heart. However, the conjugation of Acr to proteins is reversed in the renal system to free the Acr for excretion in the urine. It is likely that the Acr in urine can damage the urothelial cells in the urinary system [20, 21]. This explanation is in line with well-established findings that the Acr generated by metabolism of the antitumor drugs cyclophosphamide and isophosphamide damages the lining of the urinary system, causing blood in the urine [67, 68]

We do not observe any significant increase in BPDE-DNA adduct levels in the BAL, lung, heart, liver, or bladder mucosa in SSS-exposed mice. Given that the amount of BP is 1,000- to 10,000-fold lower than the amount of Acr in MSS, as well as in SSS, this low level of BP may be effectively “neutralized“ by components in the lining of the respiratory system, thereby considerably reducing the probability that it might damage bronchial epithelial cells. It is worth noting that the levels of Acr-dG adducts are >20 fold higher than the levels of BPDE-dG adducts in all four organs of FA-exposed control mice. Given that Acr is a byproduct of endogenous lipid peroxidation, it is likely that the Acr-dG adducts in control mice result from DNA damage induced by endogenously generated Acr [69, 70].

It has been shown that Acr-dG adducts are mutagenic, that Acr can modify repair proteins and cause inhibition in NER, BER, and mismatch repair, and that Acr treatment increases the susceptibility of human cells to DNA-dependent mutagenesis [18, 20]. The current results show that Acr can induce tumorigenic cell transformation (Fig. 5). Previously, we reported that the distribution of Acr-induced DNA adducts in the p53 gene in human bronchial epithelial cells coincides with the lung cancer p53 mutational spectrum [19]. Mutations in p53 is a major step of lung carcinogenesis; more than 40% lung cancer carry p53 mutations [31, 71]. Based on these findings we propose that Acr is a major carcinogen in MSS and SSS.

Lung cancer is the leading cause of cancer death in Taiwanese women, even though only 5% of Taiwanese women smoke [72, 73]. This high incidence of lung cancer in Taiwanese women has been attributed to two possible causes: exposure to second hand smoke and to cooking fumes [73, 74]. More than 50% of Taiwanese men smoke and women in households with smokers have more than 1.8- (in childhood) and 2.2- (adult life) fold higher incidence of lung cancer than those who live in households without smokers [75, 76]. The cooking fumes in Taiwan's kitchens are rich in Acr [74]. Women who live in households without fume extractors in the kitchen have a 6–7 fold higher incidence of lung cancer than women who live in houses with fume extractors [74, 77]. Our results suggest that induction of mutagenic Acr-dG adducts, inhibition of DNA repair, and an increase of mutational susceptibility are the three mechanisms that cause the higher incidence of lung cancer in Taiwanese women.

In summary, we found that SSS induces mainly mutagenic Acr-dG adducts in BAL, lung tissue, and bladder mucosa. SSS also inhibits DNA repair, and causes repair protein degradation in lung tissue. Since SSS is rich in Acr and Acr also can cause the same effects as SSS does, we propose that the Acr is a major component of SSS that causes lung and bladder cancer.

## MATERIALS AND METHODS

### Exposure of mice to SSS

Male FVBN mice (8-week-old, Charles River, *n* = 9–10/group/time point) were exposed to either filtered air (FA) or side-stream smoke (SSS) for 6 h/day 5 days/week for 8 or 16 weeks. The cigarette smoke (CS) was generated with an automated cigarette-smoking machine (CH Technologies, Westwood, NJ), using 2R4F cigarettes [Cigarette and Health Research Institute; stored at 24°C and 60% relative humidity (RH)]. One cigarette was lighted at a time to produce mainstream smoke (MSS) with an automatically regulated piston pump using a two-second puff of 35 ml volume once per minute. The SSS produced from the cigarette was swept up and diluted by FA and introduced into a 1.3-m^3^ stainless steel chamber for animal exposure. Mice were exposed, whole body, by inhalation, in their own cages, with food and water removed during the 6-h daily exposure period. Control mice were housed in identical exposure chambers using the same chamber flow characteristics except that the mice were exposed to charcoal-and-HEPA-filtered air. The detail of SSS exposure method is the same as described previously [78]. The average concentration of SSS was 454 ± 120 μg/m^3^ for the 8-week exposure and 454 ± 110 μg/m^3^ for the 16-week exposure. It should be noted that worldwide, in indoor environments where people smoke, the mean levels of respirable suspended particles range from 24 to 1,947 μg/m^3^ (IARC Mono vol. 83). Mice were sacrificed 24 h after the last exposure. BAL was collected by rinsing the lung with 1.2 ml of phosphate buffered saline without calcium and magnesium (Invitrogen, Carlsbad, CA) [79]. BAL was stored on ice until analysis. The lung, heart, liver, and bladder were collected and snap frozen at −80°C.

### Genomic DNA and cell lysates preparation from BAL, lung, heart, liver, and bladder-mucosa

For DNA adduct analysis, genomic DNAs were prepared from BAL, lung, heart, liver, and bladder mucosa as previously described [20]. For *in vitro* DNA repair assay, cell lysates were prepared from lung tissues of mice exposed to SSS and FA [19, 20, 23].

### DNA adduct analysis

Cyclic propano-dG adducts that formed in the genomic DNA isolated from BAL, lung, bladder-mucosa, heart, and liver of mice exposed to FA and SSS for 8 and 16 weeks were analyzed by the immunochemical methods previously described [18, 20]. PdG adduct levels in lung tissues were also analyzed by the ^32^P post-labeling 2D-TLC/HPLC method [18–20]. In brief, 10 μg of genomic DNA were digested with micrococcal nuclease and spleen phosphodiesterase (Sigma-Aldrich, St Louis, MO, USA). Samples were then digested further with nuclease NP1, and then labeled with ^32^P-γ-ATP. The resultant monophosphate nucleotides were separated by 2D-TLC, the adducted spots were eluted and then further analyzed by HPLC. The amount of adducted nucleotides was calculated based on the ^32^P ratio of adducted DNA adduct peak with the dGMP of known concentration labeled with ^32^P-γ-ATP exactly the same as in the digested genomic DNA. Given the small amount of mouse lung tissue, lung tissues were pooled from three randomly chosen mice from the same treatment group for a single chemical analysis.

### DNA repair activity and repair protein detection

The NER and BER activity in cell lysates isolated from lung tissues was determined by the *in vitro* DNA damage-dependent repair synthesis method, as described previously [18–20]. In brief, pUC18 plasmid DNAs were irradiated by UVC (1500 J/m^2^) or modified with H_2_O_2_ (100 mM, 30 min at 37°C) and used as substrates for determining the NER and BER activity, respectively. Cell lysates were isolated from lung tissues of mice exposed to FA and SSS. The assays were carried out in a mixture of substrate DNA (pUC18) and control DNA (undamaged pBR322), cell lysates, dCTP, dGTP, dTTP and ^32^P-α-dATP. The mixtures were incubated at 30°C for 3 h. The plasmid DNAs were purified and then digested with restriction enzyme HindIII. The resultant DNAs were separated by electrophoresis and the amounts of repair synthesis were detected by the radioautography. The DNA repair capacity was not determined in bladder mucosa due to the fact that the amount of cell lysates isolated from bladder mucosa was insufficient to assay DNA repair *in vitro*. The method to detect the repair proteins XPC and hOGG1 was described previously [18, 20].

### Acr-induced anchorage-independent soft-agar growth

Immortalized human bronchial epithelial cells BEAS-2B and immortalized human urothelial cells UROtsa were treated with 2.5 μM Acr for 1 h and seeded (50,000 cells per 6-cm dish) in soft-agar growth medium and incubated at 37°C for 5–6 weeks.
